# Bank complexity and core competence of commercial banks in Vietnam: The buffer role of corporate social responsibility

**DOI:** 10.1371/journal.pone.0330723

**Published:** 2025-09-05

**Authors:** Hung Quang Phung, Khoa Dang Duong, Phuc Huu Truong, Anh Quynh Pham, Hoa Thanh Phan Le

**Affiliations:** 1 Faculty of Finance and Banking, Ton Duc Thang University, Ho Chi Minh City, Vietnam; 2 School of Finance and International Business, Saxion University of Applied Sciences, Deventer, Netherlands; 3 Department of Finance, Feng Chia University, Taichung, Taiwan; 4 Faculty of Accounting and Auditing, Van Lang University, Ho Chi Minh City, Vietnam; Massey University, NEW ZEALAND

## Abstract

Based on stakeholder theory, socially responsible practices may help firms gain competitive advantages by lowering operational costs and enhancing their core competencies. From the perspective of the conglomerate hypothesis, we further suggest that CSR can act as a mechanism to reduce hidden risks, as firms, particularly banks, tend to diversify their activities more extensively. Using a sample of 26 commercial banks in Vietnam from 2010 to 2023, we test a set of hypotheses and find that engaging in CSR and diversifying operations are associated with lower risk exposure, greater financial stability, and improved asset quality. Moreover, the positive effects are more evident in large banks. In contrast, smaller banks may incur relatively higher costs when combining CSR efforts with operational complexity, potentially offsetting the benefits. Our study underscores the role of CSR as a foundation for income diversification and core competence enhancement, offering practical implications for policymakers in emerging countries to foster sustainable development in the banking sector.

## 1. Introduction

With the dual role of financial intermediation and contribution to economic progress, banks play a crucial role in the financial system. Moreover, the core competencies of banks are essential for fostering economic growth by ensuring a stable, efficient, and transparent banking system. In this context, enhancing banks’ core competencies and performance through diversification strategies and corporate social responsibility (CSR) efforts presents both a challenge and a valuable opportunity for further development. Facing serious environmental issues, Vietnam is a developing country heavily reliant on mineral resources [1]. As a response, the Vietnamese Government has issued regulations to promote a green economy. Specifically, the Prime Minister’s Decision No. 403/QD-TTg (2014) approved the National Action Plan on Green Growth.

Regarding the legal basis for implementing CSR in Vietnam’s banking sector, current regulations remain principle-based [2]. For example, the State Bank of Vietnam issued Directive No. 03/2015/CT-NHNN (2015) to encourage the expansion of green credit and control environmental and social risks in credit granting activities, as well as Decision No. 1124/QD-NHNN (2022) to promote banking finance in low-carbon production and consumption sectors [3]. In addition, according to the Director of the Legal Department of the State Bank of Vietnam, inter-ministerial coordination and sectoral collaboration are essential to assess CSR’s legal feasibility further. This trend is expected to establish a more concrete legal framework for implementing CSR in the Vietnamese banking sector.

Enhancing the core competencies of banks is essential to support national economic development. To achieve this, banks must build strong reputations and credibility, which can be reinforced through active engagement in corporate social responsibility (CSR) initiatives [4]. According to Luu et al. [5], increased bank complexity can strengthen core competencies, as diversification strategies often provide strategic advantages for banks. Similarly, Shao et al. [6] found that banks engaging in CSR tend to exhibit greater diversification in their operations, assuming that CSR serves as a mechanism for implicit risk mitigation. These efforts contribute to risk reduction, improved stability, better asset quality, and enhanced reputations, collectively building core competencies. On the other hand, Zhou et al. [7] pointed out that fulfilling CSR obligations can impose specific costs on banks, especially under short-term resource constraints. This issue may increase pressure on expenditures such as human resource training. DeYoung and Roland [8] also noted that expanding into non-interest income activities may generate higher fixed costs due to required technology and human capital investments, which could ultimately diminish banks’ core competencies. AlKhouri and Arouri [9] further argued that CSR engagement and increased bank complexity may lead to costs that outweigh potential benefits, particularly when bank executives must operate beyond their expertise and core functions.

Previous studies on bank complexity and the impact of CSR engagement on banks’ core competencies have primarily focused on developed economies, such as the United States, China, and the United Kingdom. However, limited attention has been given to developing countries. However, no studies have examined this relationship using data from these markets. Therefore, further empirical research is needed in emerging contexts such as Vietnam. The first research question explores whether findings from developed countries remain consistent when applied to data from developing economies. Investigating the role of income diversification in Vietnamese commercial banks is essential for several reasons. First, Vietnam is a rapidly growing economy undergoing significant financial reforms; thus, understanding the effects of income diversification can help evaluate the financial system’s stability and resilience. Second, the country’s reliance on sectors such as real estate and fossil fuels highlights the importance of income diversification in identifying potential risks and new growth opportunities. Third, empirical evidence from Vietnam contributes to the literature on frontier markets by offering more profound insights into the relationship between bank performance and income diversification. The second research question examines whether CSR engagement moderates or mediates the relationship between income diversification and the core competencies of commercial banks in Vietnam. We adopt the two-stage least squares (2SLS) approach to address this research question, following Fan et al. [10] and Bawono and Handika [11] to mitigate endogeneity concerns. Our analysis is based on a panel dataset of 26 Vietnamese commercial banks covering 2010–2023. To ensure the robustness of our results, we perform sub-sample analyses based on bank size and apply robustness checks, including panel-corrected standard errors (PCSE) and robust least squares (RLS), to control for potential issues such as multicollinearity, heteroscedasticity, and autocorrelation [12].

Furthermore, this study makes significant contributions both theoretically and practically. In terms of theoretical contribution, the study builds an integrated framework between corporate social responsibility (CSR), bank complexity (ID), and core competence (CC), thereby filling the research gap, as previous literature mainly focuses on developed markets or isolated aspects such as green finance. Unlike prior studies, this study emphasizes the systemic role of CSR in mitigating risks arising from diversification strategies, especially in the context of banks in emerging economies such as Vietnam. Regarding practical relevance, the research results help policymakers and bank leaders better understand how CSR can support risk management and improve operational efficiency. At the same time, the study provides strategic directions for banks in building a sustainable development model, effectively leveraging core competencies and controlling risks associated with organizational complexity. This contribution is relevant to the Vietnamese financial market, which is transforming substantially and moving towards deeper international integration. In addition, fundamental theories such as neoclassical economics, stakeholder theory, agency theory, and the conglomerate hypothesis provide essential theoretical foundations for the topic. They help explain conflicting views on the impact of CSR, clarify the role of CSR in reducing governance risks, moderating self-interested behavior, and improving operational efficiency when banks pursue a diversification strategy. Integrating these theories enables the study to build a rigorous research model appropriate to the context of Vietnamese banks [13].

The study is structured as follows. Section 2 outlines the theoretical framework. Section 3 provides a comprehensive literature review and presents the development of the hypotheses. Section 4 describes the research methodology, including data collection, econometric modeling, and estimation techniques. Section 5 presents the empirical results. Section 6 discusses the key findings. Finally, Section 7 concludes the study.

## 2. Literature review

### 2.1. The relationship between CSR and core competence

There are several explanations for why CSR efforts positively influence core competencies. According to stakeholder theory, it is assumed that profitability and engaging in CSR activities are not mutually exclusive and that acting in the best interests of all stakeholders can ultimately lead to improved organizational performance [14]. Firms that engage in socially responsible activities may gain a competitive advantage by attracting more investors and customers while reducing operating costs [15]. Additionally, Belasri et al. [16] argue that bank performance and CSR are positively associated. They claim that CSR initiatives can help organizations build a strong reputation, which brings several benefits, such as a greater ability to attract and retain high-quality employees. Furthermore, Wu and Shen [4] also found that CSR positively impacts banks’ financial performance (FP). They argue that when banks engage in large-scale CSR, customers may be more willing to accept lower interest rates on their deposits; from the banks’ perspective, lower-cost deposits represent lower-cost funding sources. Outstanding CSR performance and the resulting reputation enhancement can foster customer loyalty and attract clients from competitors, thereby increasing market share [16]. Kim et al. [17] also found that CSR efforts improve a firm’s financial performance, particularly when competition among firms is intense. Therefore, banks engaging in CSR activities may derive various benefits, including increased appeal to customers and investors, reduced cost of capital, enhanced brand value, and improved financial outcomes [15].

In contrast, neoclassical economic theory argues that funds allocated to CSR are inefficient. They should be redirected toward initiatives that maximize corporate value, and banks’ performance may decline due to CSR engagement [16,18,19]. Zhou et al. [7] argue that banks incur specific costs when fulfilling their social responsibilities due to short-term resource limitations. Banks that overinvest in CSR programs may undermine their core operations. Therefore, firms with limited resources tend to allocate their funds more prudently, investing only in CSR initiatives that are deemed essential and serve to reduce internal and external conflicts [1]. In addition, CSR can be beneficial, particularly for organizations operating under resource constraints. Nguyen and Nguyen [1] found that banks with abundant resources may engage in excessive CSR spending or treat CSR participation as more discretionary, which could increase the risk of failure rather than ease stakeholder tensions.

Previous studies examining the relationship between CSR participation and banks’ core competencies have focused on developed countries such as China and the United Kingdom. Our study selects a sample of commercial banks in Vietnam because the banking sector is pivotal in economic development and financial reform. Furthermore, the institutional environment in Vietnam is still evolving, with many banks facing challenges related to transparency, risk management, and reputation building. Proactive CSR implementation enhances trust among customers, investors, and shareholders and strengthens internal capabilities such as human resource management, operational efficiency, and innovation capacity. Therefore, we propose the following hypothesis:

Hypothesis 1. There is a positive relationship between CSR and the core competence of Vietnam commercial banks.

### 2.2. The relationship between bank complexity and core competencies

The conglomerate hypothesis argues that diversification of banking activities ensures that managerial efforts are optimized across different areas of banking operations [20]. This trend leads to economies of scope by sharing fixed costs across various products and reducing bank earnings volatility. Specifically, by offering a broad range of products and services, banks can utilize their existing infrastructure, resources, and customer base more efficiently. This approach can result in cost savings, improved operational efficiency, and enhanced profitability. In addition, Luu et al. [5] found that banks’ operational performance and income diversification are positively correlated. They argue that adopting this diversification strategy can generate substantial benefits for banks, including increased profitability, economies of scope, reduced monitoring costs through shared fixed expenses, better utilization of managerial capabilities, risk mitigation via expansion into multiple products and regions, enhanced franchise value and market resilience, and improved ability to build competitive advantage when entering new markets.

In contrast, agency theory [21,22] suggests that firms should concentrate on their core activities to minimize potential agency problems. It contends that diversification may lead managers to expand their firms beyond an optimal scale, undermining firm value. Moreover, diversification may not always be beneficial, as its associated costs can sometimes outweigh its advantages. When bank managers operate outside their core competencies and areas of expertise, it can lead to negative consequences such as increased information asymmetry, inefficient allocation of resources, higher agency costs, intensified competition, and a decline in competitive advantage [9]. Due to the inherent uncertainty of non-traditional banking activities, income diversification may increase the overall risk profile of banks. DeYoung and Roland [8] explained that weaker customer-bank relationships in non-lending sectors are a key reason why revenues from non-traditional businesses tend to be more volatile than those from conventional banking services. Additionally, engaging in non-interest activities may require substantial investments in technology and human capital, leading to significantly higher operational costs and increased earnings volatility.

Previous studies on the relationship between income diversification and banks’ core competencies have focused on developed countries like the United States. Our study selected a data sample from a developing country like Vietnam because its banking sector is currently undergoing a transitional phase and is deeply influenced by ongoing economic restructuring. In the context of rising competition and shrinking profit margins from traditional credit activities, banks are increasingly compelled to seek alternative revenue streams from services, investments, and retail finance. Therefore, banks must enhance their core competencies to mitigate operational risks and avoid losing competitive advantage, especially in an institutional environment that is still evolving, as is the case in Vietnam. Therefore, we propose the following hypothesis:

Hypothesis 2. There is a positive relationship between income diversification strategy and the core competence of Vietnam commercial banks.

### 2.3. The buffering role of CSR on the relationship between bank complexity and core competencies

The conglomerate hypothesis demonstrates diversification’s benefits but highlights its risks, particularly when increasing complexity leads to ineffective management. The study by Luu et al. [5] expands upon the hypothesis by incorporating CSR as a buffering mechanism that helps mitigate the adverse effects of complexity on core competencies. Furthermore, Shao et al. [6] found that banks diversify their operations more extensively under the assumption that CSR functions as an implicit form of risk mitigation. They also found evidence that banks can reduce risk, enhance stability, and improve asset quality by broadening their activities and engaging in CSR initiatives. CSR activities may include investments in socially responsible projects, adoption of sustainable business practices, or initiatives that address social and environmental challenges. Through these efforts, banks can strengthen their reputation, foster stronger relationships with stakeholders, and lower their exposure to risks associated with non-compliance, reputational harm, or mismanagement. This approach, in turn, can contribute to improved asset quality as the bank’s overall risk profile becomes more balanced and sustainable [8].

Conversely, AlKhouri and Arouri [9] suggest that engaging in CSR amid growing bank complexity involves costs that may outweigh the benefits, primarily when executives must operate outside their core competencies and areas of expertise. Chiaramonte et al. [23] also provide evidence that diversification is not universally advantageous for banks, particularly when it involves unfamiliar industries or regions. Additionally, diversification can lead to greater risk and reduced stability if the associated costs surpass the potential gains [24]. Moreover, Lahouel et al. [25] demonstrate that overinvestment can negatively and significantly affect banks’ financial health, regardless of the income diversification measure used. Banks with high CSR performance and ambitious income diversification strategies may incur additional costs, resulting in reduced investment in training and development necessary to strengthen core competencies [24,26].

Previous studies exploring the relationship between income diversification, CSR participation, and the core competencies of banks have primarily been conducted in major economies such as the United States and China. Our study selected a data sample from a developing country like Vietnam because it is a transitional economy where the banking sector must expand its operations while simultaneously meeting sustainable development demands and improved transparency. In the context of shrinking profits from traditional credit activities, Vietnamese banks are increasingly diversifying their income sources to support growth, yet this also heightens complexity and operational risk. Effective risk control and maintaining core competencies remain significant challenges as the legal framework evolves. In such an environment, CSR is a complementary mechanism to build reputation, mitigate risks, and enhance internal cohesion. Therefore, we propose the following hypothesis:

Hypothesis 3. Corporate social responsibility plays an essential buffering role in the positive relationship between income diversification and the core competencies of banks in Vietnam.

## 3. Data and methodology

### 3.1. Data

The study collected data from 26 commercial banks in Vietnam from 2010 to 2023. All the commercial banks’ financial statements are among the trustworthy databases. Having been established as a policy bank entirely funded by state funds, the analysis excludes Agribank following Duong et al. [3]. The study sampling period is from 2010 to guarantee no effects on the estimations from the global financial crisis of 2008. We winsorize the sample at the 1^st^ and 99^th^ percentiles to exclude outlier issues and follow Duong et al. [3] and Tran et al. [27] to remove observations for which the data is insufficient to calculate the necessary variables. The final sample is an unbalanced panel of 358 annual observations from 26 commercial banks in Vietnam.

### 3.2. Variable definitions

#### 3.2.1. *Core competence.*

We follow Luo et al. [28] and Duong et al. [3] to compute a bank core competence comprehensive index, a new way to measure bank performance. To achieve this, we apply principal component analysis (PCA) to a selected set of variables, including return on assets, return on equity, non-interest income ratio, non-performing loans ratio, cost-to-income ratio, loan-to-deposit ratio, capital adequacy ratio, provision coverage ratio, net interest margin, asset growth rate, deposit growth rate, market share of deposits, loan market share, and loan growth rate. The results of the PCA, which reflect the core competence of banks, are presented in S1 Appendix.

#### 3.2.2. *Corporate social responsibility.*

Our method of disclosure follows Duong et al. [3], Duong et al. [29] and Duong et al. [30]. We analyze CSR disclosure into five major sectors: society, environment, employees and customers, suppliers, and products [31,32]. The range of the CSR index is 0–5 [31,32].

#### 3.2.3. *Bank complexity.*

While the prior study by Duong et al. [3] only focused on CEO power and green credit, this paper follows Vuong and Nguyen [33] to introduce the concept of bank complexity. This proxy is measured through income diversification to represent the extent to which banks expand their operations beyond traditional sources of income. This approach emphasizes that bank complexity is an important mediating factor affecting the relationship between CSR and core competencies, which helps to expand the analysis compared to the previous papers that only focused on the direct impact of CSR. In addition, while the study by Le-Bao [34] mainly focused on the effects of diversification strategy on bank technical efficiency through a DEA model, this study inherits and extends it in several important directions. First, we shift the focus from technical efficiency to core competence, a more comprehensive indicator reflecting banks’ long-term performance and competitiveness. Second, instead of only considering the role of ownership and bank age, we include corporate social responsibility (CSR) as a strategic moderating mechanism in the relationship between income diversification and core competence. Overall, these extensions allow us to provide a more comprehensive and policy-relevant perspective on how CSR and diversification strategy jointly shape the long-term competitiveness of banks.

We use income diversification (ID) to measure bank complexity. We follow Nguyen et al. [2] and Luu et al. [5] to take one minus HHI. The Herfindahl-Hirschman index, or HHI of income specialization, is determined by splitting total operational income into its non-interest and net interest income components.


$$HHI=\;\left[{\left(\frac{NII}{NOI}\right)}^{2}+\;{\left(\frac{NONII}{NOI}\right)}^{2}\right]$$

Net interest income is represented by the acronym NII, non-interest income by NONII, and net operational income by NOI. As the HHI increases, the bank gets less varied and more consolidated [35]. HHI has a range of 0–1. In the end, the study arrived at the following income diversification calculation:


$$ID=1-HHI=1-\;\left[{\left(\frac{NII}{NOI}\right)}^{2}+\;{\left(\frac{NONII}{NOI}\right)}^{2}\right]$$

This measurement method has more clearly reflected the bank’s income structure change and the potential risks when banks increase their diversification. The higher ID value indicates that banks have higher diversification banking activities. The advantage of this method is that it can be measured comprehensively by reflecting the bank’s income structure in Net Interest Income and Non-Interest Income activities.

### 3.3. *Model construction*

According to the stakeholder theory, Belasri et al. [16] and Wu and Shen [4] discovered a favorable correlation between banks’ performance and their implementation of CSR. In contrast, Nguyen and Nguyen [2] and Zhou et al. [7] argued that CSR endeavors can harm banks’ performance in line with neoclassical economic theory. Therefore, this study builds Model (1) to test hypothesis 1 to see how CSR effectiveness affects the core competencies of banks in Vietnam and test stakeholder theory and neoclassical economic theory.


$${CC}_{i,t}\;=\;\beta_{0}\;+\;\beta_{1}{CSR}_{i,t}\;+\;\sum \beta_{q}{Control}_{i,t}\;+\;\alpha_{i}\;+\;\alpha_{t}\;+{\;\mu }_{it}$$ (1)

Regarding bank complexity, Luu et al. [5] found evidence that the conglomeration hypothesis explains why income diversity enhances bank performance. However, due to the agency theory, Ammar and Boughrara [22] and Nguyen [36] argued that complexity negatively influences bank performance. Thus, this study constructed Model (2) to examine how income diversification (ID) affects core competence.


$${CC}_{i,t}\;=\;\beta_{0}\;+\;\beta_{1}{ID}_{i,t}\;+\;\sum \beta_{q}{Control}_{i,t}\;+\;\alpha_{i}\;+\;\alpha_{t}\;+{\;\mu }_{it}$$ (2)

In addition, this study constructed the 3^rd^ Model to determine whether banks’ core competencies are impacted by CSR performance and bank complexity in the same Model.


$${CC}_{i,t}\;=\;\beta_{0}\;+\;\beta_{1}{CSR}_{i,t}+\beta_{2}{ID}_{i,t}+\;\sum \beta_{q}{Control}_{i,t}\;+\;\alpha_{i}\;+\;\alpha_{t}\;+{\;\mu }_{it}$$ (3)

To further test for stakeholder theory and the buffering role of CSR on the relationship between income diversification and core competence of Vietnamese commercial banks, we add the interaction variable (CSR*ID) to the regression model (4), following the results of Harjoto and Laksmana [24], Shao et al. [6], AlKhouri and Arouri [9], and Luu et al. [5].


$${CC}_{i,t}\;=\;\beta_{0}\;+\;\beta_{1}{CSR}_{i,t}\;+\;\beta_{2}{ID}_{i,t}\;+\;\beta_{3}{CSR}_{i,t}\;*{\;ID}_{i,t}\;+\;\sum \beta_{q}{Control}_{i,t}\;+\;\alpha_{i}\;+\;\alpha_{t}\;+\;\mu_{it}$$ (4)

Where “i” is cross-sections, “t” is time, and “α” is the intercept. In addition, CSR represents the corporate social responsibility index; ID is income diversification; CC represents core competence. Control variables include the non-performing loans (NPL), bank size (SIZE), bank tangible assets (TANG), and return on assets ratio (ROA). α_i_ is the firm fixed effect, and α_t_ is the year fixed effect. _it_ is the residual value. S2 Appendix has definitions for all variables.

### 3.4. Estimation methodology

The study used the Hausman and Redundant tests to determine which of the three estimate techniques – Pooled Ordinary Least Squares (OLS), Fixed Effects Model (FEM), and Random Effects Model (REM) – was the most suitable. Ordinary Least Squares (OLS) estimates linear regression parameters by minimizing squared prediction errors. It is efficient and straightforward under classical assumptions but can be violated by multicollinearity, heteroscedasticity, or endogeneity problems. FEM controls unobserved heterogeneity by allowing individual-specific intercepts, effectively reducing omitted variable bias, but it can be less efficient and cannot estimate time-invariant variables. The REM postulates that each effect is independent of the regressors and random, providing more efficient estimates if assumptions hold, but can be biased if the effects are correlated with any regressors. However, according to Greene [37], endogeneity issues are violated by OLS, FEM, and REM. As a result, we employ the Durbin-Wu-Hausman test by Duong et al. [3] to check for endogenous problems. Lastly, the study applies the Two-Stage Least Squares (2SLS) approach following Fan et al. [10] and Bawono and Handika [11] to address the endogenous variables. According to Wang et al. [38], the Two-Stage Least Squares (2SLS) estimate approach handles endogeneity in regression models by using instrumental variables. In parallel with using the primary method of 2SLS to overcome the endogeneity problem, we have combined it with fixed effects to eliminate estimation bias due to unobserved factors related to the independent and dependent variables [39]. Combining GLS with cross-section weights ensures that units with high variability do not distort the regression results, improving heteroscedasticity [40]. On the other hand, in panel data, changes across periods can cause serial correlation or time-varying variance. Period covariance weights help mitigate this phenomenon, ensuring more accurate estimates of standard errors [41].

Furthermore, the research carried out two robustness tests to validate the consistency of our primary empirical outcomes. First, we perform a robustness test by splitting our data into two subgroups: small banks and major banks, based on the State Bank of Vietnam’s Circular 52/2018/TT-NHNN. The second robustness test re-estimates the main regression result using the Panel-Corrected Standard Errors (PCSE) and the Robust Least Squares (RLS) method.

## 4. Empirical results

### 4.1. Descriptive statistics

**Fig 1:**
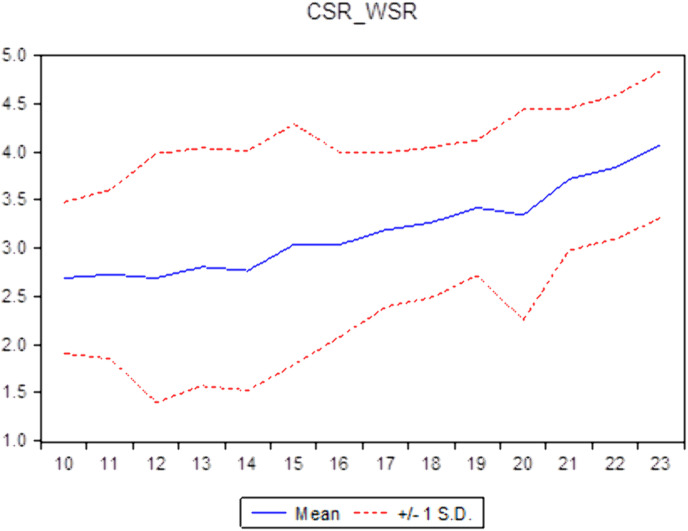
The Mean and ±1 Standard Deviation of CC of commercial banks in Vietnam (source: author’s calculations).

**Fig 2:**
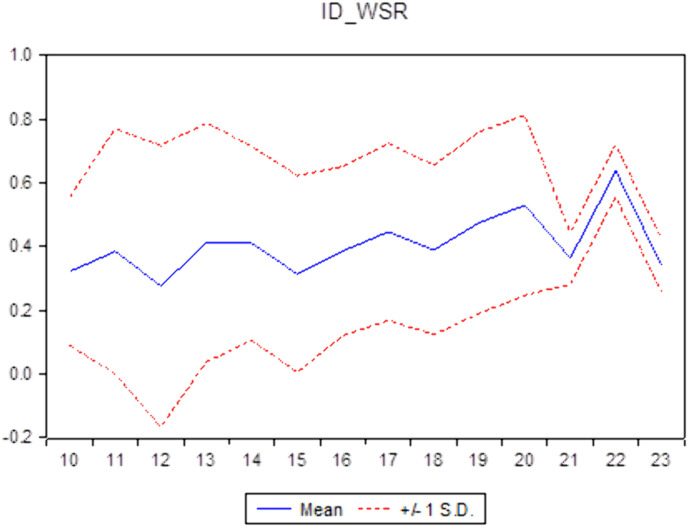
The Mean and ±1 Standard Deviation of CSR of commercial banks in Vietnam (source: author’s calculations).

**Fig 3:**
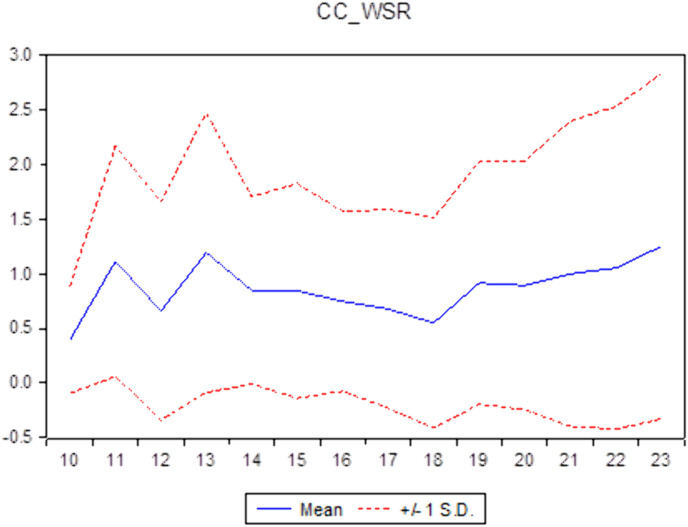
The Mean and ±1 Standard Deviation of ID of commercial banks in Vietnam (source: author’s calculations).

Descriptive statistics help researchers understand the general characteristics of the data, thereby identifying the appropriate direction to address the research question. Descriptive statistics not only assist in verifying the reliability of the data but also provide a solid foundation for subsequent quantitative analyses, ensuring coherence and logical consistency throughout the research process. Table 1 illustrates the descriptive statistics of the sample. It shows a standard deviation of roughly 1.0613 and the 0.8763 mean value of CC. The average CSR is 3.1704, similar to findings from the study [29] in Vietnam. The ID’s average value of approximately 0.4053 aligns with the average ID of Vietnamese commercial banks, which was 0.201 for 11 years from 2007 to 2017 [5]. This result means that commercial banks in Vietnam have increased their income diversification in recent years. Additionally, compared to the countries of the Gulf Cooperation Council, Vietnamese commercial banks have a lower ID than [9,42] and higher than that of all 14 economies in the Asia Pacific [43]. Table 1 shows that the non-performing loans (NPL) have a 0.0201 mean value and a 0.0135 standard deviation value. The descriptive statistics of other variables, including the bank size (SIZE), tangible assets (TANG), and return on assets (ROA) of the banks, are also shown in this table. Figs 1–3 report the CC, CSR, and ID trends of commercial banks in Vietnam during the sampling period.

**Table 1:** Descriptive statistics.

	Mean	Median	Maximum	Minimum	Std. Dev.	Observations
CC	0.8763	0.8739	3.7178	−2.5002	1.0613	358
CSR	3.1704	3.0000	5.0000	0.0000	1.0433	358
ID	0.4053	0.3906	1.2901	−0.1552	0.2948	358
NPL	0.0201	0.0184	0.0878	0.0000	0.0135	358
SIZE	32.5491	32.6024	35.2854	30.3192	1.1998	358
TANG	0.1421	0.0091	2.2911	−0.3288	0.4640	358
ROA	0.0095	0.0076	0.0356	0.0001	0.0073	358

Note: Table 1 provides the descriptive statistics for all variables used in the analysis. The dataset includes 358 observations from 26 Vietnamese commercial banks from 2010 to 2023. Detailed definitions of all variables can be found in S2 Appendix.

### 4.2. Pearson correlation matrix

The independent variables’ correlations are displayed in Table 2. Notably, CSR exhibits a strong positive correlation with TANG, presented by a coefficient equal to 0.2902, and ROA, which is presented by a coefficient equal to 0.2401, indicating a significant relationship with these factors. Consequently, we computed the variance inflation coefficient (VIF) and discovered that multicollinearity issues do not exist because all VIF values are less than five [27].

**Table 2:** Pearson correlation matrix.

	CSR	ID	NPL	SIZE	TANG	ROA	VIF
CSR	1						1.1484
	-----						
ID	0.0269	1					1.2107
	(0.612)	-----					
NPL	0.3244***	0.0863	1				1.0625
	(<0.001)	(0.103)	-----				
SIZE	−0.0731	−0.0908*	−0.0457	1			1.3623
	(0.168)	(0.086)	(0.388)	-----			
TANG	0.2902***	0.3574***	0.3477***	−0.1841***	1		1.1200
	(<0.001)	(<0.001)	(<0.001)	(<0.001)	-----		
ROA	0.2401***	−0.0502	0.6319***	0.0530	0.1876***	1	1.1747
	(<0.001)	(0.344)	(<0.001)	(0.317)	(<0.001)	-----	

Note: Table 2 presents the Pearson correlation coefficients for all variables utilized in the study. The dataset comprises 26 Vietnamese commercial banks observed from 2010 to 2023. Detailed definitions of all variables are provided in S2 Appendix. Statistical significance levels are indicated by ***, **, and * for the 1%, 5%, and 10% thresholds, respectively.

### 4.3. Estimation results

Based on the results in Table 3, the REM method was selected for Model (1) based on the Hausman test results, with Prob (Hausman Test) equal to 0.471 (less than 0.05), not enough basis to reject the hypothesis H0, showing that REM is more suitable than FEM. On the contrary, Models (2), (3), and (4) have Prob (Hausman Test) of 0.028, 0.001, and 0.001 (less than 0.05), respectively, rejecting the hypothesis H0, so FEM was selected. At the same time, the Redundant Fixed Effect Test in all models has Prob less than 0.001, confirming that FEM is more suitable than the pooled Model.

**Table 3:** Regression results from the REM and FEM methods.

Variable	Model (1)	Model (2)	Model (3)	Model (4)
	REM	FEM	FEM	FEM
CSR	0.3366***		0.4378***	0.3601***
	(<0.001)		(<0.001)	(<0.001)
ID		1.6269***	1.7483***	1.1729***
		(<0.001)	(<0.001)	(0.001)
CSR*ID				0.2055*
				(0.070)
NPL	5.5665	5.1381	4.3588	4.1309
	(0.108)	(0.116)	(0.113)	(0.132)
SIZE	0.0226	0.1730	0.1332	0.1199
	(0.713)	(0.127)	(0.162)	(0.207)
TANG	−0.0740	−0.2681	−0.2964	−0.2929
	(0.447)	(0.356)	(0.224)	(0.228)
ROA	50.8243	66.0569	58.1624	58.5463
	(<0.001)	(<0.001)	(<0.001)	(<0.001)
Constant	−1.5071	−6.1117*	−6.1506**	−5.5044*
	(0.444)	(0.098)	(0.047)	(0.077)
Number of orbs	358	358	358	358
R-squared	0.2708	0.6352	0.7377	0.7405
Adjusted R-squared	0.2604	0.5854	0.7009	0.7030
F-statistic	26.1379	12.7556	20.0089	19.7810
Prob(F-statistic)	(<0.001)	(<0.001)	(<0.001)	(<0.001)
Prob (Redundant Test)	(<0.001)	(<0.001)	(<0.001)	(<0.001)
Prob (Hausman Test)	0.471	0.028	0.001	0.001
Durbin-Watson stat	1.2677	0.9300	1.0526	1.0375
Prob (Breusch-Pagan)	(<0.001)	(<0.001)	(<0.001)	(<0.001)
Prob. (Wald test)	(<0.001)	(<0.001)	(<0.001)	(<0.001)

Note: Table 3 presents the regression outcomes based on both Fixed Effects Model (FEM) and Random Effects Model (REM) estimations. The analysis uses a panel dataset of 26 Vietnamese commercial banks from 2010 to 2023. Definitions of all variables can be found in S2 Appendix. Statistical significance is denoted by ***, **, and *, corresponding to the 1%, 5%, and 10% levels, respectively.

In terms of the Model’s explanatory power, R-squared shows that FEM is superior to REM. Specifically, the R-squared increased from 0.6352 in Model (2) to 0.7405 in Model (4), and the Adjusted R-squared also improved from 0.5854 to 0.7030, demonstrating the effectiveness of adding the interaction variable CSR*ID. The F-statistic values of all models have Prob (F-statistic) less than 0.001, indicating that the models are overall statistically significant. FEM, especially Model (4), is not only selected through appropriate tests but also has superior explanatory power through the R-squared and F-statistic indices.

After applying FEM and REM method to analyze the effect of ID and CSR on CC, the study uses the Durbin-Wu-Hausman test, as described by Duong et al. [29], to determine whether endogenous variables exist. Model (5) is initially built as the endogenous examination test model:


$${CSR}_{i,t}\;=\;\beta_{0}\;+\;\beta_{1}{ID}_{i,t}\;+\;\beta_{2}{NIM}_{i,t}+\;\beta_{3}{NPL}_{i,t}+{\;\beta }_{4}{SIZE}_{i,t}+\;\beta_{4}{TANG}_{i,t}+\;\alpha_{i}+\;\alpha_{t}+\;\mu_{it}$$ (5)

As an alternative variable of CSR, the CSR residuals (Res-CSR) obtained in the first step are included in the model (3) [3]. An endogeneity issue exists if the residual’s coefficient is a statistically significant variable. We also test for ID, NPL, SIZE, TANG, and ROA.

Table 3 shows that the probability of the Wald test is less than 0.05, indicating a heteroscedasticity problem. Table 4 shows that endogenous variables exist in the models, so we follow Fan et al. [10], Bawono and Handika [11] to perform the 2SLS combined with cross-sectional weights to ensure that highly volatile units do not bias the regression results, improving the problem of heteroscedasticity [40]. At the same time, Table 3 detected autocorrelation issues, so we added period covariance weights to help adjust the Model to address this phenomenon, ensuring more accurate estimates of standard errors [41]. The 2SLS estimations are reported in Table 5.

**Table 4:** Coefficient of residual variables.

Residual of variables	Coefficient	P-value
Res_CSR	0.4400***	(<0.001)
Res_ID	2.4618***	(<0.001)
Res_NPL	−5.0397*	(0.098)
Res_SIZE	0.3055***	(<0.001)
Res_TANG	0.2794***	(0.003)
Res_ROA	78.8474***	(<0.001)
C	0.8763***	(<0.001)

Note: Table 4 shows that the residuals of CSR, ID, SIZE, and ROA are statistically significant, indicating that these variables are dependent. This finding suggests that the endogenous variables in the Model include CSR, ID, NIM, and TANG. The levels of statistical significance are denoted by ***, **, and * for the 1%, 5%, and 10% thresholds, respectively.

**Table 5:** Regression results from the 2SLS.

Variable	Model (1)	Model (2)	Model (3)	Model (4)
CSR	0.3366***		0.3886***	0.2828***
	(<0.001)		(<0.001)	(<0.001)
ID		1.4370***	1.5683***	0.7950***
		(<0.001)	(<0.001)	(0.008)
CSR*ID				0.2605***
				(0.005)
NPL	5.5665	5.4330**	2.7257	2.6565
	(0.124)	(0.042)	(0.252)	(0.262)
SIZE	0.0226	0.0804	−0.1595**	−0.1791***
	(0.723)	(0.231)	(0.012)	(0.006)
TANG	−0.0740	0.1341	0.1553*	0.1809**
	(0.492)	(0.126)	(0.057)	(0.031)
ROA	50.8243***	33.8061***	26.7684***	26.8690***
	(<0.001)	(<0.001)	(<0.001)	(<0.001)
Constant	−1.5071	−2.7807	3.8712*	4.8172**
	(0.461)	(0.200)	(0.054)	(0.019)
Number of orbs	358	358	358	358
R-squared	0.2708	0.7214	0.7624	0.7686
Adjusted R-squared	0.2604	0.6959	0.7398	0.7458
F-statistic	26.1379	28.3121	33.7363	33.7353
Prob(F-statistic)	(<0.001)	(<0.001)	(<0.001)	(<0.001)
GLS weight	Cross-section weight	Cross-section weight	Cross-section weight	Cross-section weight
Coefficient covariance	Period weight	Period weight	Period weight	Period weight
Durbin-Watson stat	1.2677	1.2089	1.2316	1.2322

Note: Table 5 presents the estimation results obtained using the Two-Stage Least Squares (2SLS) method. The analysis is based on a panel dataset of 26 Vietnamese commercial banks from 2010 to 2023. Detailed descriptions of all variables are provided in S2 Appendix. Statistical significance is indicated by ***, **, and *, corresponding to the 1%, 5%, and 10% levels, respectively.

### 4.4. Robustness tests

Nguyen and Nguyen [1] argued that firms with limited resources allocate their funds more prudently, investing just in CSR initiatives that are deemed vital and serving as conflict-reduction tools. At the same time, financially unconstrained banks may overinvest in CSR initiatives, harming the bank’s financial performance. In addition, Ammar and Boughrara [22] stated that more prominent banks frequently take an aggressive approach to diversification, which causes problems with their managers’ agencies. Therefore, Model (4) was used in the research to investigate the robustness of our primary findings using subsamples of varying bank sizes found in Table 6. According to Circular 52/2018/TT-NHNN issued by the State Bank of Vietnam, commercial banks are classified into two primary categories based on their total assets. These categories encompass large banks with total assets exceeding VND 100,000 billion and small banks with total assets of VND 100,000 billion or less.

**Table 6:** Regression results from across bank size subsamples.

Variable	Full sample	Small banks	Large banks
CSR	0.2828***	0.5235***	0.3149***
	(<0.001)	(<0.001)	(<0.001)
ID	0.7950***	2.4269***	0.8535**
	(0.008)	(<0.001)	(0.016)
CSR*ID	0.2605***	−0.0345	0.2188**
	(0.005)	(0.854)	(0.039)
NIM	2.6565	14.7407***	−4.1865**
	(0.262)	(<0.001)	(0.031)
NPL	−0.1791***	−0.7023*	−0.3398***
	(0.006)	(0.066)	(<0.001)
SIZE	0.1809**	0.0963	0.1652*
	(0.031)	(0.757)	(0.063)
TANG	26.8690***	54.3451	24.9578***
	(<0.001)	(<0.001)	(<0.001)
C	4.8172**	20.6001	10.0055***
	(0.019)	(0.106)	(<0.001)
Number of orbs	358	114	244
R-squared	0.7686	0.8592	0.9134
Adjusted R-squared	0.7458	0.8035	0.9003
F-statistic	33.7353	15.4412	69.59
Prob(F-statistic)	(<0.001)	(<0.001)	(<0.001)
Durbin-Watson stat	1.2322	1.3329	1.2399

Note: Table 7 represents the robustness test results from 2SLS regression. The sample consists of 26 Vietnamese commercial banks from 2010 to 2023. All variable definitions are reported in S2 Appendix. The expression of significance at 1%, 5%, and 10% is shown by ***, **, and *, respectively.

Then, the study implemented the Two-Stage Least Squares (2SLS) method with different bank sizes to verify the stability of our main findings through model 4. When comparing the factors affecting a bank’s core competence, one can see that its scale affects its financial capacity, operational network, and business strategy. Large banks often have more substantial resources and investment capabilities, helping them develop core competencies more effectively. Classifying by size helps us know how banks of different sizes will be affected by various factors [12].

Finally, we estimate Model 4 results by using three different regression methods, including the Two-Stage Least Squares (2SLS), the Panel-Corrected Standard Errors (PCSE), and the Robust Least Squares (RLS) to affirm the consistency of our main findings. Alfadli et al. [44] suggested that the PCSE method is used to analyze models with balanced panel data, helping to control serial correlation and cross-sectional dependence between variables. Thus, it generates more accurate results on the impact of industry-specific, macroeconomic, and bank-specific elements on bank performance. Costa et al. [45] suggested that the RLS method solves the problem of data uncertainty and lack of reliability in prediction and improves the accuracy and robustness of semi-supervised image classification. Furthermore, Wang et al. [38] claim that this method simultaneously minimizes the influence of noise and uneven data structure. It also optimizes the parameter estimation process and enhances classification capabilities.

## 5. Discussion

Table 5 shows the regression results using the 2SLS method. CSR significantly improves the core competencies of banks. In particular, the core competency index increases by approximately 0.2828 points for each increase in the CSR index. Our results are consistent with the results of Wu and Shen [4], Siueia et al. [15], and Belasri et al. [16], who suggest that CSR efforts can positively affect the core competencies of banks. On the input side, by adopting a CSR strategy, banks can improve their competitive advantage by attracting and retaining valuable employees, increasing employee productivity and loyalty, and promoting teamwork and enthusiasm for their work [10]. Therefore, a decrease in the cost of deposits equates to a lower input cost for the bank, improving the bank’s financial performance. On the output side, Belasri et al. [16] argued that CSR in banks can generate many benefits, including increased customer reputation and loyalty, increased market share by attracting customers from competitors, increased trust and attractiveness of investors, lenders, and depositors, which may lead to improved long-term financial performance of commercial banks [17]. However, our results are inconsistent with those of Belasri et al. [16], Zhou et al. [7], and Nguyen and Nguyen [1] because these studies used data samples from countries with the most developed economies, such as the United States and China. This finding supports the stakeholder theory but does not support the neoclassical economic theory because this theory suggests that funds allocated to CSR are wasteful. These financial resources optimize firm value so that the performance of banks will be affected by the application of CSR. Our results also support hypothesis 1.

As shown in Table 5, bank complexity strengthens the core competencies of banks in Vietnam. Specifically, our findings show that increasing income diversification by one point increases the CC index by 0.7950 points. Our findings are consistent with those of Luu et al. [5]. According to Luu et al. [5], implementing an income diversification strategy has the potential to increase bank profitability and economic scope, reduce monitoring costs, improve the use of managerial expertise, minimize the risk through expansion across multiple products and geographies, improve franchise value and market beta, and strengthen the ability to increase competitive advantage when entering new markets. However, our results are inconsistent with AlKhouri and Arouri [9] and DeYoung and Roland [8] because these studies use data samples from developed countries such as the UK. Therefore, the results are inconsistent with our results. Our findings support the conglomerate hypothesis but not the agency theory, which argues that firms should focus on their traditional activities to avoid potential agency problems. They assert that diversification may encourage managers to grow their firms beyond their ideal size at the expense of their value. Our results support hypothesis 2.

Table 5 reports the significant buffering role of CSR on the positive association between income diversification and the core competencies of banks. Banks that increase complexity and engage in CSR activities will increase their core competencies by 0.2605 points. In other words, when banks increase their complexity by implementing an income diversification strategy, their core competencies will increase, and the increase in CSR performance will strengthen this relationship. Our results are consistent with the findings of Shao et al. [6] and DeYoung and Roland [8]. Banks that engage in more CSR activities will significantly increase the diversity of their banking activities. Banks can reduce their risk exposure, become more stable, and improve their asset quality by diversifying their activities and engaging in CSR activities. This finding supports the notion that CSR is an implicit risk-hedging technique. As banks become more complex and diversified, managers are more likely to pursue power than shareholder-benefit projects [22]. Furthermore, as banks become more complicated, they tend to engage in aggressive diversification strategies, which come with costs that may outweigh the benefits and negative impacts that arise as bank executives are forced to work beyond their core competencies and experience [5]. However, our results are inconsistent with AlKhouri and Arouri [9] and Harjoto and Laksmana [24] because these studies used data samples from economically developed countries like the United States. Hence, our results are inconsistent with their results. Our findings support the conglomerate hypothesis and hypothesis 3.

Table 5 reports that bank size (SIZE) significantly negatively affects banks’ core competence. Our results align with Ammar and Boughrara [22] and Nguyen and Nguyen [1]. According to Nguyen and Nguyen [1], banks with unlimited resources might overinvest in CSR projects or engage in more optional activities, raising the risk of bank failure rather than reducing stakeholder tensions. In addition, Ammar and Boughrara [22] argued that since managers are more likely to enter new business sectors to grow their influence and take advantage of perks rather than create shareholder value, more complicated and well-known banking institutions tend to engage in aggressive diversification initiatives. This trend might result in agency problems.

Table 5 illustrates that tangible assets (TANG) positively impact CC. Our result supports the result of Nawaz [46], who argues that physical assets combined with human capital can leverage a bank’s core competencies. This trend helps the bank convert ideas into tangible assets, increasing operational efficiency. However, our results conflict with those of Donnellan et al. [47], who argue that tangible assets can negatively affect core competence if they become obsolete or the firm over-invests in them at the expense of developing critical intangible assets such as innovation and employee skills.

Table 5 also shows that ROA has a positive impact on CC. Our results are consistent with those of Phan et al. [48], who demonstrated that ROA is a key performance indicator supporting core competencies development through income diversification. They argue that ROA is a core indicator of asset utilization, reflecting the bank’s profitability and the success of resource management. Income diversification increases ROA by expanding non-interest income sources, strengthening core competencies, and reducing financial risks.

Tables 6 and 7 provide the robustness test results. Table 6 reports that the positive influences of implementing CSR and income diversification strategy on banks’ core competence and the empowerment role of CSR on the relationship between bank complexity and core competence remain unchanged across bank size subsamples. Moreover, the main findings are robust after employing alternative estimation methods such as the PCSE and the RLS.

**Table 7:** Robustness test results using alternative estimation methods.

Variable	2SLS	PCSE	RLS
CSR	0.2828***	0.3601***	0.2395***
	(<0.001)	(<0.001)	(<0.001)
ID	0.7950***	1.1729***	1.2588***
	(0.008)	(<0.001)	(<0.001)
CSR*ID	0.2605***	0.2055**	0.2611**
	(0.005)	(0.039)	(0.039)
NPL	2.6565	4.1309	3.8456
	(0.262)	(0.117)	(0.117)
SIZE	−0.1791***	0.1199	−0.1356
	(0.006)	(0.228)	(0.228)
TANG	0.1809**	−0.2929	0.1096
	(0.031)	(0.221)	(0.221)
ROA	26.8690***	58.5463***	46.5177***
	(<0.001)	(<0.001)	(<0.001)
C	4.8172**	−5.5044*	3.1700*
	(0.019)	(0.089)	(0.089)
Number of orbs	358	358	358
R-squared	0.7686	0.7405	0.4793
Adjusted R-squared	0.7458	0.7030	0.4689
F-statistic	33.7353	19.7810	
Prob(F-statistic)	(<0.001)	(<0.001)	
Durbin-Watson stat	1.2322	1.0375	

Note: Table 6 represents the robustness test results from RLS regression. The sample consists of 26 Vietnamese commercial banks from 2010 to 2023. All variable definitions are reported in S2 Appendix. The expression of significance at 1%, 5%, and 10% is shown by ***, **, and *, respectively.

## 6. Conclusion

### 6.1. Conclusion

This study investigates how CSR and bank complexity develop the core competence of commercial banks in Vietnam. The study employs the 2SLS approach to examine a sample from 26 commercial banks in Vietnam from 2010 to 2023. This study yields some noteworthy findings. The findings indicate that the bank’s core competency is improved by strengthening its CSR efforts. Secondly, commercial banks that are more complex in income diversification increase their core competence. Third, by developing CSR activities and implementing an income diversification strategy, commercial banks can strengthen their core competence by reducing the potential risks of aggressively engaging in diversification strategies, such as agency problems, since CSR is an implicit hedging strategy and a control mechanism. These findings are robust across size subsamples. The findings remain robust even if we employ alternative estimation methods such as PCSE and RLS. Stakeholder theory, the conglomeration hypothesis, and earlier research align with the study, even if it is inconsistent with agency and neoclassical economic theories.

### 6.2. Implication

The empirical findings support the bank managers and policymakers in emerging markets in developing sustainable banking performance in the following ways. First, we will make recommendations to stakeholders based on our research results. Shareholders should consider income diversification and CSR initiatives as value-added strategies because income diversification helps stabilize customer profits, while CSR helps enhance reputation and long-term sustainability, benefiting shareholders. Customers will often expect banks to participate in socially conscious activities. Banks providing more diverse and stable services through income diversification and CSR will give customers a positive view of more stable and customer-oriented services. Employees should see banks’ CSR efforts as a positive signal of ethical and responsible business because income diversification helps build a more stable and safe working environment for employees.

Second, we will make some recommendations to policymakers. In encouraging and monitoring CSR and income diversification, they should develop and strengthen the legal framework to promote and monitor the implementation of CSR and income diversification of banks, which will help create a more sustainable and responsible banking industry. In the current context in Vietnam, CSR regulations are only general principles, and further assessment of legal feasibility is needed. Coordination between ministries and sectors to build a specific legal framework on CSR in the banking sector is extremely necessary. This policy relates to the balance of risks in income diversification; the authorities should issue guidelines to ensure the balance between income diversification and risk control and equip banks with prudent risk management measures to avoid excessive risk-taking. They can issue policies to encourage banks to implement CSR, develop preferential policies such as tax exemptions and reductions for banks that effectively implement CSR projects, or provide financial support or preferential loan programs to promote CSR activities, create a healthy competitive environment, and encourage responsible and diversified banking operations. Policymakers need to improve the legal framework on CSR in Vietnam further, develop specific regulations on the minimum budget ratio for CSR, and develop clear criteria to evaluate CSR effectiveness in the banking industry. In addition, banks need to establish clear risk management plans when expanding into new business areas, especially for high-risk industries such as financial investment and insurance. The role of the State Bank is also vital in implementing this process; they need to play a coordinating and technical support role for banks to implement CSR effectively and sustainably, as well as coordinate with ministries and branches to ensure the legality and feasibility of applying CSR.

Finally, the responsibility belongs to the leading players, such as commercial banks; we suggest that they adjust policies based on the size and type of bank. Applying CSR at the strategic level is essential for large banks to manage risks in diverse and complex operations while creating long-term competitive advantages. These banks need to integrate CSR into their overall business strategy, focusing on projects with significant impact, such as environmental protection, community development, and green technology investment. CSR should be used as a risk management tool, especially in complex areas such as financial investment, insurance, and international banking services. Meanwhile, small banks should prioritize CSR at the community level to improve their image and build trust with customers and partners. They can focus on local support activities such as education, health, infrastructure development, and charity programs to develop sustainable relationships with the community. CSR can also become an effective marketing tool, helping to enhance its reputation and increase competitiveness in the domestic market. The most essential thing for banks is human capital; they need to train and improve the capacity of members of their organization. Commercial banks should regularly develop training programs to enhance the capacity to implement CSR for senior managers. Bank managers should implement intensive training programs to improve CSR strategy planning skills, including impact assessment, risk management, and measuring the effectiveness of CSR initiatives. Banks must also disseminate international standards and organize regular workshops to update global CSR trends and relevant legal changes. In short, both policymakers and stakeholders play an essential role in promoting these activities, contributing to building a sustainable, stable, and socially responsible banking system. In addition, integrating CSR enables banks to build a reputable image and enhance trust among customers and the broader community, strengthening core competencies such as brand reputation and service quality. A diversification strategy allows banks to reduce concentration risk, expand growth opportunities, and develop new capabilities. When CSR and diversification are effectively integrated, banks achieve short-term performance gains and establish a strong foundation for long-term competitive advantage through enhanced internal capabilities and greater adaptability.

### 6.3. Limitation

Although this study extends the prior literature on CSR and diversification in the banking industry, it has the following drawbacks. Firstly, the study is constrained by a relatively modest sample size of only 358 annual observations. This limitation is attributed to data restrictions and may require more robust statistical inferences. Some Vietnamese commercial banks need the data to create CSR and core competence indices covering 2010–2023. As a result, observations with incomplete data were not included in the research analysis. Second, given that Vietnam is a frontier and transitional market, the study’s conclusions have limited relevance to rising and industrialized nations. With these limitations in mind, the study emphasizes how important it will be to broaden the scope of its data to enable a comparative evaluation of CSR performance and the diversified income influence on banks’ core competence in developed and developing countries. This strategy will produce more thorough insights.

## Supporting information

S1 Appendix The result of the Principal Component Analysis for bank core competence. (DOCX)

S2 Appendix Variable definitions. (DOCX)
