# Supplementary material for: Bank complexity and core competence of commercial banks in Vietnam: The buffer role of corporate social responsibility
Source: PLoS One. 2025 Sep 5;20(9):e0330723. doi: 10.1371/journal.pone.0330723 (PMC12413003; doi:10.1371/journal.pone.0330723)
Supplement: S1 Appendix — (DOCX) [file pone.0330723.s001.docx]

| **Appendix A:** The result of the Principal Component Analysis for bank core competence. | | | | | | | | | | | | | | | |
| --- | --- | --- | --- | --- | --- | --- | --- | --- | --- | --- | --- | --- | --- | --- | --- |
| Eigenvalues: (Sum = 15, Average = 1) | | | | | | | | | | | | | | | |
| Number | 1 | 2 | 3 | 4 | 5 | 6 | 7 | 8 | 9 | 10 | 11 | 12 | 13 | 14 | 15 |
| Value | 2.978 | 2.349 | 1.875 | 1.490 | 1.243 | 1.053 | 0.874 | 0.738 | 0.613 | 0.523 | 0.423 | 0.341 | 0.272 | 0.155 | 0.067 |
| Difference | 0.629 | 0.286 | 0.385 | 0.217 | 0.135 | 0.210 | 0.179 | 0.135 | 0.125 | 0.090 | 0.100 | 0.081 | 0.069 | 0.117 | --- |
| Proportion | 0.198 | 0.156 | 0.125 | 0.099 | 0.082 | 0.070 | 0.058 | 0.049 | 0.040 | 0.034 | 0.028 | 0.022 | 0.018 | 0.010 | 0.004 |
| Cumulative Value | 2.978 | 5.327 | 7.202 | 8.692 | 9.936 | 10.989 | 11.861 | 12.603 | 13.216 | 13.740 | 14.163 | 14.504 | 14.777 | 14.932 | 15 |
| Cumulative Proportion | 0.198 | 0.355 | 0.480 | 0.579 | 0.662 | 0.732 | 0.790 | 0.840 | 0.881 | 0.916 | 0.944 | 0.967 | 0.985 | 0.995 | 1 |
| Eigenvectors (loadings): | | | | | | | | | | | | | | | |

| Variable | PC 1 | PC 2 | PC 3 | PC 4 | PC 5 | PC 6 | PC 7 | PC 8 | PC 9 | PC 10 | PC 11 | PC 12 | PC 13 | PC 14 | PC 15 |
| --- | --- | --- | --- | --- | --- | --- | --- | --- | --- | --- | --- | --- | --- | --- | --- |
| ROAA | 0.2931 | 0.3542 | -0.0254 | 0.2415 | -0.0587 | 0.0632 | -0.381 | 0.4401 | 0.0785 | -0.251 | 0.1867 | -0.1412 | 0.0657 | -0.5312 | 0.0115 |
| ROAE | 0.4213 | 0.2028 | 0.0702 | 0.1983 | 0.0156 | -0.0851 | -0.2714 | 0.189 | -0.1533 | -0.1214 | -0.194 | 0.0567 | 0.1134 | 0.7235 | -0.0507 |
| % Non-interest Income | -0.0182 | -0.0985 | 0.1054 | 0.1573 | -0.2025 | 0.7854 | -0.3701 | -0.1656 | -0.0952 | 0.2795 | 0.0367 | 0.0654 | -0.1123 | 0.0415 | -0.0152 |
| Cost-to-income ratio | -0.3125 | -0.1693 | -0.0754 | -0.1834 | 0.0791 | 0.0857 | 0.0542 | 0.7082 | -0.2837 | 0.2274 | 0.261 | 0.1856 | 0.1273 | 0.1506 | 0.011 |
| Loan-to-deposit ratio | 0.3605 | -0.0502 | -0.2094 | 0.1503 | 0.0172 | 0.0443 | 0.3474 | 0.2651 | 0.0387 | 0.5173 | -0.3351 | -0.3135 | -0.3291 | -0.0702 | 0.1247 |
| Current asset ratio | 0.2821 | 0.0813 | -0.3617 | -0.4285 | -0.0105 | 0.0168 | -0.1513 | -0.0246 | 0.125 | 0.0965 | -0.2714 | 0.6574 | 0.0231 | -0.1698 | 0.0125 |
| Core capital adequacy ratio | -0.0487 | 0.4123 | -0.3265 | 0.0402 | -0.0162 | 0.2125 | 0.2465 | -0.0251 | 0.5871 | 0.1458 | 0.3672 | -0.0243 | 0.1803 | 0.2749 | 0.015 |
| NPL ratio | -0.2183 | -0.0665 | -0.1983 | 0.186 | 0.5408 | 0.3805 | 0.1189 | 0.1541 | 0.1213 | -0.4103 | -0.4407 | 0.0153 | 0.022 | -0.0101 | 0.0154 |
| Provision coverage | 0.0614 | 0.2025 | 0.1711 | -0.208 | -0.5654 | 0.3035 | 0.4971 | 0.1704 | -0.1312 | -0.393 | -0.1583 | 0.0791 | -0.0206 | 0.0134 | 0.0101 |
| Net interest margin | 0.1731 | 0.3512 | -0.1427 | 0.2205 | 0.3127 | 0.0885 | 0.3242 | -0.2813 | -0.5841 | 0.0502 | 0.2965 | 0.2215 | -0.0482 | -0.118 | 0.0121 |
| Asset growth rate | -0.0835 | -0.0592 | 0.3442 | 0.6251 | -0.1101 | -0.1825 | 0.1762 | 0.0831 | 0.2291 | 0.1287 | -0.0815 | 0.5481 | -0.0246 | -0.0971 | 0.017 |
| Deposit growth rate | -0.0108 | 0.2849 | 0.4497 | -0.2985 | 0.3171 | 0.0618 | -0.0564 | 0.0925 | 0.155 | -0.0214 | 0.104 | 0.0773 | -0.664 | 0.095 | 0.0273 |
| Loan growth rate | 0.0134 | 0.3281 | 0.4702 | -0.2245 | 0.199 | 0.0887 | 0.0663 | -0.0428 | -0.0304 | 0.3205 | -0.2914 | -0.1173 | 0.5801 | -0.1441 | 0.0204 |
| Market share of deposits | 0.4015 | -0.3742 | 0.165 | -0.0954 | 0.1468 | 0.1156 | 0.1075 | -0.0215 | 0.105 | -0.1632 | 0.2471 | 0.0725 | 0.1517 | 0.0023 | 0.6984 |
| Market share of loan | 0.3982 | -0.3598 | 0.1375 | -0.0867 | 0.1594 | 0.1194 | 0.1813 | 0.0394 | 0.1471 | -0.0658 | 0.2153 | 0.0235 | 0.0845 | -0.0552 | -0.7048 |
