# Supplementary material for: Bank complexity and core competence of commercial banks in Vietnam: The buffer role of corporate social responsibility
Source: PLoS One. 2025 Sep 5;20(9):e0330723. doi: 10.1371/journal.pone.0330723 (PMC12413003; doi:10.1371/journal.pone.0330723)
Supplement: S2 Appendix — (DOCX) [file pone.0330723.s002.docx]

| **Appendix B:** Variables definition | | | |
| --- | --- | --- | --- |
| Variables | Notation | Variable definition | References |
| Dependent variable | | | |
| CC | Core competence | Commercial bank core competency index | Duong et al. (2023), Luo et al. (2021) |
| Independent variables | | | |
| CSR | Corporate Social Responsibility | The four components of the CSR disclosure index are the following: environment, society, workers and products, customers, and suppliers. | Duong et al. (2023). |
| ID | Income diversification | One minus H.H.I.; $HHI= \left[ \left( \frac{NII}{NOI} \right)^{2}+ \left( \frac{NONII}{NOI} \right)^{2} \right]$ | Nguyen et al. [35] |
| Control variables | | | |
| NPL | Non-performing Loans | The ratio of non-performing to gross loans | Duong et al. (2022) |
| SIZE | Bank size | Total assets' natural logarithm | Duong et al. (2023) |
| TANG | Tangible assets | The resources consist of tangible elements, including equipment, machinery, and knowledgeable staff. | Donnellan et al. (2019) |
| ROA | Return on assets | This ratio equals profit after tax over average total assets. This ratio measures how effectively a business is using its assets to generate profits. | Phan et al. (2022) |
